# Estrogen and Androgen Blockade for Advanced Prostate Cancer in the Era of Precision Medicine

**DOI:** 10.3390/cancers10020029

**Published:** 2018-01-23

**Authors:** Tetsuya Fujimura, Kenichi Takayama, Satoru Takahashi, Satoshi Inoue

**Affiliations:** 1 Department of Urology, National Center for Global Health and Medicine, Tokyo 162-8655, Japan; tfujimura-jua@umin.ac.jp; 2 Department of Functional Biogerontology, Tokyo Metropolitan Institute of Gerontology, Tokyo 173-0015, Japan; ktakayama-tky@umin.ac.jp; 3 Department of Urology, Nihon University School of Medicine, Tokyo 173-8610, Japan; takahashi.satoru@nihon-u.ac.jp

**Keywords:** prostate cancer, androgen receptor, estrogen-related receptor, stem cell, androgen deprivation therapy (ADT), selective estrogen receptor modulators (SERMs), personalized medicine, precision medicine

## Abstract

Androgen deprivation therapy (ADT) has been widely prescribed for patients with advanced prostate cancer (PC) to control key signaling pathways via androgen receptor (AR) and AR-collaborative transcriptional factors; however, PC gradually acquires a lethal phenotype and results in castration-resistant PC (CRPC) during ADT. Therefore, new therapeutic strategies are required in clinical practice. In addition, ARs; estrogen receptors (ERs; ERα and ERβ); and estrogen-related receptors (ERRs; ERRα, ERRβ, and ERRγ) have been reported to be involved in the development or regulation of PC. Recent investigations have revealed the role of associated molecules, such as *KLF5*, *FOXO1*, *PDGFA*, *VEGF-A*, *WNT5A*, *TGFβ1*, and *micro-RNA 135a* of PC, via ERs and ERRs. Selective ER modulators (SERMs) have been developed. Recently, estrogen and androgen blockade (EAB) using a combination of toremifene and ADT has been demonstrated to improve biochemical recurrence rate in treatment-naïve bone metastatic PC. In the future, the suitability of ADT alone or EAB for individuals may be evaluated by making clinical decisions on the basis of information obtained from RT-PCR, gene-panel, or liquid biopsy to create a “personalized medicine” or “precision medicine”. In this review, we summarize ER and ERR signaling pathways, molecular diagnosis, and SERMs as candidates for advanced PC treatment.

## 1. Introduction

Prostate cancer (PC) is the second-most frequently diagnosed cancer in men worldwide, with 1.1 million new cases estimated to have occurred in 2012 [1]. PC is the fifth leading cause of death due to cancer worldwide, with the highest mortality rates reported in the Caribbean (29.3 per 100,000) and Southern (24.4 per 100,000) and Middle Africa (24.2 per 100,000) [1]. Mortality rates due to PC have decreased in most of the developed countries, including those in the North America, Oceania, and Northern and Western Europe [1]. In contrast, mortality rates have increased in some Asian and Central and Eastern European countries, such as Korea, China (Hong Kong), and Russia. Chemoprevention, prostate-specific antigen (PSA) screening for early detection, and innovative treatments for advanced PC are necessary to reduce the resultant mortality due to PC.

Systematic treatment for advanced or metastatic PC includes androgen deprivation therapy (ADT) or chemotherapy. Conventional ADT involved surgical or medical castration and the administration of anti-androgen agents, such as bicalutamide, flutamide, and nilutamide. Recently, new anti-androgen agents, such as enzalutamide and abiraterone, have been approved for castration-resistant PC (CRPC) [2]. Docetaxel and cabazitaxel are also available for CRPC. However, effects of these new agents are transitory. Moreover, they are relatively expensive, costing 10,759$ for 160 mg of enzalutamide; 9817$ for 1000 mg of abiraterone per month; 1919$ for 120 mg of docetaxel; and 10,639$ for 40 mg of cabazitaxel, respectively (https://www.drugs.com/price-guide/). To improve cancer-specific survival (CSS) and reduce the cost incurred on drugs used for advanced PC treatment, durable and economic therapeutic strategies are warranted worldwide. Here, we reviewed the literature pertaining to endocrine therapy and proposed a new therapeutic strategy involving estrogen and androgen signal blockade (EAB) for advanced PC.

## 2. Initiating Endocrine Therapy for PC

Pioneering work by Huggins [3] established diethylstilbestrol (DES) administration as a low-cost but effective treatment for metastatic PC. However, due to adverse side effects associated with DES treatment, including the exacerbation of heart failure, vascular complications, and gynecomastia, DES therapies have been replaced with the application of luteinizing hormone-releasing hormone analogs and anti-androgen agents, such as lueprolide, goserelin, bicalutamide, and flutamide, since the past two decades. The precise functional network of androgen receptors (ARs), co-factors, and micro-RNA (miR) has been clarified using next generation sequencing in these lines [4,5]. Recently, the use of selective estrogen modulators (SERMs) for advanced PC treatment has reemerged due to the discovery of various nuclear receptors and their functional analyses in context of PC.

## 3. Nuclear Receptors Associated with Endocrine Therapy for PC

Initially, the action of estrogens was believed to be mediated via the blockade of the pituitary–testicular axis, which effectively decreased circulating androgen levels and induced tumor regression. This concept was supported by immunohistochemical and in situ hybridization studies conducted in the 1990s, which could not identify any detectable estrogen receptor (ER) levels in the epithelial compartments of the human prostatic tissue [6]. The classical ER, ERα, is dominantly expressed in the stromal compartment but not in the glandular epithelium of the normal human prostate [6,7,8]. Estrogens actions on prostatic epithelium have been considered to be exerted via ERα-mediated paracrine mechanism. Conversely, the exposure of humans or rodents to estrogens induced proliferative changes and squamous metaplasia in their prostates [9,10,11]. Noble strain rats treated with androgen plus estrogens over a long period have been reported to show high PC incidence [12]. The mechanism underlying estrogen action was clarified in the past two decades owing to the successful cloning of nuclear receptors, such as ERα, ERβ, and estrogen-related receptors (ERRs: ERRα, ERRβ, and ERRγ) from 1980 to 1996 [13,14,15,16]. Evidence suggests an overlap between ERR and ER biology.

In the 1990s, the status of ERα expression in PC remained controversial [17,18,19,20,21,22,23]. ERα protein expression was more frequently observed in higher-grade metastatic cancers than in low-to-moderate grade tumors [17]. Conversely, other investigators noted the presence of ERα expression in well-differentiated adenocarcinoma but not in poorly differentiated tumors and metastatic lesions [24,25,26]. In our study, over 70% of the malignant epithelium showed no ERα expression, whereas distinct ERα immunoreactivity was identified in 7% of the cancerous loci in 50 patients with localized PC [27]. Previous researchers have reported variable expression patterns of ERα in PC [28].

Novel ERβ cloned from a rat prostate cDNA library was found to be localized in the epithelial compartment of the rat prostate [14]. ERα and ERβ are paralogs and they share 86%, 23%, 17%, and 100% amino acid identity in DNA-binding domains (DBDs), N-termini, C-termini, and ligand-biding domains (LBDs), respectively. However, because of the divergence in their LBDs, the two ER subtypes bind ligands (agonists or antagonists) with different affinities [29]. Although several studies have investigated the expression of the second ER, ERβ, in normal and malignant epithelium of the human prostate, the available data on ERβ protein expression in the human prostate remains controversial [17,18,19,20,21,22,23,30,31]. While one study reported a decrease in the ERβ protein expression in PC, another reported an increase. This discrepancy may be attributed to the presence of C-terminal truncated splice variant of ERβ, ERβl-ERβ5 [32] and the specificity of ERβ antibody [33]. The expression of ERβ3 is limited to the testis, whereas ERβ1, ERβ2, ERβ4, and ERβ5 are expressed in the prostate [32]. Anderson showed the schematic view of antibody epitopes and ERβ isoforms, and validated 13 commercially available or in-house produced ERβ antibodies [33]. Out of the 13 ERβ-targeting antibodies, PPZ0506 and 14C8 appeared to specifically target ERβ in formalin fixed paraffin embedded-treated cell lines [33].

Next, cDNA for ERRα was isolated by screening cDNA libraries with probes corresponding to the DBD of human ERα [16]. ERRα has a high homology to ERα at DBD and also recognizes estrogen response element (ERE) [34,35,36,37,38]. ERRα mRNA was highly expressed in the heart and skeletal muscle and expressed to a lesser degree in the kidney, pancreas, small intestine, and colon in humans [39]. ERRα is now established to be associated with unfavorable biomarkers in human breast cancer [40,41]. Moreover, ERRα and ERRγ are associated with unfavorable and favorable biomarkers, respectively, in human PC [42,43].

The steroid and xenobiotic receptor (SXR), also known as the human pregnane X receptor, constitutes members of the nuclear receptor superfamily of ligand-activated transcription factors [44,45]. The elimination of dihydrotestosterone (DHT), which is the active androgen in the prostate, is critical for successful endocrine therapy for PC. Testosterone is inactivated in the liver and prostate by Cytochrome P450 (CYP) enzymes [46,47,48]. SXR regulates CYP3A4 and CYP2B6, which are responsible for the hydroxylation of testosterone in the liver and prostate [48,49,50]. SXR and its target genes *CYP3A4* and *CYP2B6* participate in the regulation of PC via intra-prostatic testosterone metabolism [49,50,51,52].

## 4. Clinicopathological Features of Nuclear Receptors in PC

wtERβ expression was significantly lower in cancers than in benign epithelium and inversely correlated with the Gleason grade. In contrast, ERβcx (ERβ2) was significantly more expressed in high-grade cancers than in low-grade tumors. The CSS of patients with lower wtERβ expression was significantly worse than that of patients with higher wtERβ expression. ERβcx (ERβ2) expression was correlated with the Gleason grade and inversely correlated with EBβ expression [27]. Higher ERβcx (ERβ2) expression was significantly correlated with poor CSS.

This finding was corroborated by the results of the following investigations of ERβ splice variants. Tissue microarrays constructed using samples from 566 men who had undergone radical prostatectomy were analyzed using immunohistochemistry for wtERβ, ERβ2, ERβ5, and G-protein-coupled receptor-30 (GPR 30) [53]. Intense cytoplasmic wtERβ staining was independently associated with time to recurrence (Hazards ratio (HR) 1.7, 95% confidence interval (CI) 1.1–2.6, *p* = 0.01) and PC-specific mortality (HR 3.9, 95% CI 1.8–24.9, *p* = 0.01). Similarly, intense nuclear ERβ2 staining was independently associated with PC-specific mortality (HR 3.9, 95% CI 1.1–13.4, *p* = 0.03) [53]. Samples obtained from 100 patients with cT3N0M0 PC were evaluated to determine the expression of AR, ERα, and wtERβ as well as the clinical outcomes, including biochemical recurrence (BCR), progression-free survival (PFS), and overall survival (OS) [54]. BCR was defined with two consecutive PSA values of ≥0.2 ng/mL. Disease progression was diagnosed by digital rectal examination, computed tomography, or bone scintigraphy. AR expression was not associated with any of the above outcomes; however, patients with high ERα or low wtERβ immunoreactivity scores than those with negative ERα or high wtERβ immunoreactivity scores showed 6.03-, 10.93-, and 10.53-times greater hazards for BCR, PFS, and OS, respectively. Nuclear ERβ2 and cytoplasmic ERβ5 expression served as significant prognostic factors for BCR and PFS in 144 men with localized PC, who had undergone radical prostatectomy [54].

ERRα expression was elevated in PC, particularly in those with a higher Gleason grade, compared with benign epithelium foci, and higher ERRα expression acted as a significant prognostic predictor of CSS [42]. In contrast, ERRγ expression decreased in PC and acted as a preferable prognostic marker of PC [43].

SXR immunoreactivity was significantly lower in cancerous lesions than in the benign foci of specimens obtained from radical prostatectomy. CSS in patients with high SXR expression was significantly increased, and the combined data of SXR and CYP3A4 showed that a higher expression of SXR and CYP3A4 was correlated with better CSS [49]. In addition, CYP2B6 was abundantly localized in the cytoplasm of normal epithelial cells compared with that of PC cells. CYP2B6 immunoreactivity was inversely correlated with higher Gleason grade. Patients with decreased CYP2B6 expression showed poor CSS [51]. CYP2B6 overexpression in LNCaP cells significantly decreased testosterone-induced proliferation. Thus, SXR, CYP3A4, and CYP2B6 may regulate PC progression via testosterone metabolism.

## 5. Functional Analysis of Nuclear Receptors and Associated Factors for Endocrine Therapy

**Figure 1:**
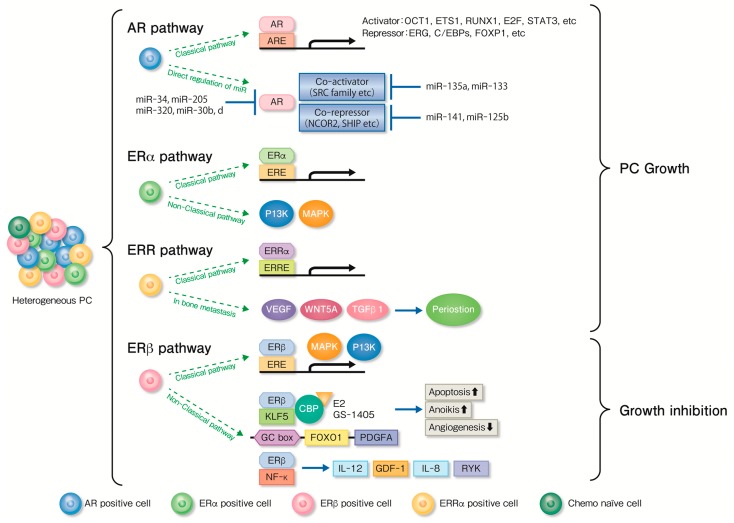
Nuclear receptors associated with prostate cancer progression including androgen receptor (AR), estrogen receptor (ER), and estrogen-related receptor (ERR).

We summarize the pathway of the nuclear receptors associated with endocrine therapy for PC (Figure 1). In the classical pathway, ERα binds to EREs to regulate its gene transcription [55]. ERα also participates in several non-classical pathways, including ERE-independent gene transcription via protein–protein interactions with transcription factors, and rapid non-genotropic pathways. ERα functions have been investigated in two mouse models of aggressive PC: Phosphatase and Tensin Homolog (PTEN)-deficient and Hi-MYC mice [56]. ERα promoted cell proliferation in PTEN-deficient tumors by regulating phosphatidylinositol-3 kinase (PI3K) and mitogen-activated protein kinase (MAPK) signaling pathways and glucose sensitivity.

Various genes of cell proliferation and bone metastasis gens are known to be recruited by ERβ [57]. E2 imparts paradoxical effects in PC because E2 biphasically regulates prostate tumor growth by suppressing *Forkhead Box O1* (*FOXO1*) and *Platelet Derived Growth Factor Subunit A* (*PDGFA*) expression levels through ERβ and Kruppel Like Factor (KLF) 5 pathways [58,59]. E2 treatment decreased KLF5-dependent *FOXO1* transcription in PC cells through ERβ, inhibiting apoptosis and increasing tumor weight in mouse xenograft models [59]. In contrast, when mice were treated with higher doses of E2, prostate tumor growth was suppressed through ERβ and KLF5 pathways. In fact, E2 inhibited *PDGFA* transcription and suppressed angiogenesis through ERβ and KLF5 pathways. PDGFA recovered angiogenesis inhibited by E2. Both *PDFGA* and *FOXO1* expressions were markedly suppressed by higher doses of E2, and angiogenesis was insufficient for prostate tumor growth, leading to the suppression of tumor growth [59]. ERβ and NFκB also regulate PC activating *Interleuikin (IL)-12*, *Growth differentiation gene (GDF)-1*, *IL-8*, and *Receptor-like tyrosine kinase (RYK)* [60].

ERβ activation is a target for treating early stage PC to prevent cancer progression [61,62]. RNA sequencing and immunohistochemistry were conducted to compare gene expression profiles in the ventral prostate of young (2-month-old) and aging (18-month-old) ERβ/ERβ mice and their wild-type littermates. ERβ modulates AR signaling by repressing AR driver RORc and increasing AR co-repressor dachshund family (DACH 1/2). ERβ loss resulted in the upregulation of genes whose expression is associated with poor prognosis in PC, accompanied with the downregulation of tumor-suppressive or tumor-preventive genes. ERβ agonist (LY3201) treatment resulted in the nuclear import of PTEN and repression of AR signaling.

ERβ activation is also a treatment option for CRPC [62]. The influence of the ERβ-specific ligand 8β-VE2 was investigated using three kinds of VCaP cells through pretreatment, under ADT, and under maximum ADT. 8β-VE2 treatment reduced the overexpression of AR as well as AR splice variants (ARVs) lacking the ligand binding domain in VCaP cells under maximum ADT.

ERRα has been reported to promote several traits of cancer progression, such as proliferation [63,64,65], epithelial–mesenchymal transition [66], resistance to hypoxia [67], angiogenesis [68], and cell migration [69] in prostate, breast, or colon cancer. ERRα was also involved in the bone tumor progression of CRPC [70]. Increased ERRα levels in tumor cells led to rapid tumor progression, with both bone destruction and formation accompanied with osteoclasts and osteoblasts. *Vascular Endothelial Growth Factor A* (*VEGF-A)*, *Wnt Family Member 5A* (*WNT5A)*, and *Transforming Growth Factor Beta 1 (TGFβ1)* were upregulated by *ERRα* expression in tumor cells. In addition, *ERRα* regulated tumor stromal microenvironment by stimulating pro-metastatic factor *periostin* expression. With no natural ligands of ERRα involved miR-135a, modulated ERRα function [71,72]. miR-135a downregulated ERRα expression through specific sequences of its 3′-untranslated region (UTR) and also decreased the cell invasive potential of ERRα pathway [72].

## 6. SERMs for PC

SERMs are involved in major therapeutic advancements in clinical practice for breast cancer, osteoporosis, and PC. The first-generation clomiphene, the second-generation toremifene and raloxifene, and the third-generation ospemifene and bazedoxifene have been used in diseases affecting women [73]. SERMs are economical, costing 90$ for 50 mg of clomiphene; 1313$ for 60 mg of toremifene; and 16$ for 60 mg of raloxifene per month, respectively. SERMs are synthetic ligands for ERs that can exhibit either estrogenic or anti-estrogenic effects, depending on the tissue type [73]. SERMs have tissue-specific agonist–antagonist activity [74]. For example, raloxifene exhibited diverse activities via ER depending on ERα or ERβ expression in the target organ [75]. When ERE-luciferase (ERE-LUC) and either ERα or ERβ were co-transfected into HEK 293 cells, toremifene acted as a potent antagonist of the 17β-estradiol-stimulated transactivation of ERα (at 1 μM) and ERβ (at 5 μM), respectively [74]. In contrast, toremifene (0.1 μM) served as a potent antagonist of ERα (95% inhibition), but not of ERβ (20% inhibition). Thus, toremifene is a more selective antagonist of ERα, the activation of which is implicated in prostate epithelial growth, than of ERβ [74]. Toremifene significantly reduced PC incidence in a transgenic adenocarcinoma of mouse prostate model [76]. Toremifene treatment resulted in a significant reduction in prostate tumor growth and PC3M (a human PC cell line) proliferation, expressing ERα [77].

Details of clinical investigations of SERMs are summarized in Table 1 [78,79,80,81,82]. A phase II trial of toremifene was conducted in 15 patients with CRPC [78]. The patients received toremifene at a dose of 300 to 600 mg/m^2^, which is a relatively high dosage. The treatment was well-tolerated and toxicity was mild; however, no objective responses were achieved [78]. Another study used toremifene at a dose of 20 to 60 mg to prevent PC incidence in men with high-grade prostatic intraepithelial neoplasia (HGPIN) [79]. A total of 514 patients with HGPIN and no PC evidence on screening biopsy were randomized to 20, 40, or 60 mg toremifene dose or to a placebo daily for 12 months. The patients underwent re-biopsy at 6 and 12 months. The cumulative risk of PC was decreased in patients on 20 mg toremifene compared with that in placebo controls (24.4% vs. 31.2%, *p* < 0.05). The annualized rate of prevention was 6.8 cancers/100 treated men. In patients with no biopsy evidence of cancer at baseline and at 6 months, the 12-month incidence of PC was decreased by 48.2% with 20 mg toremifene as compared with the placebo controls (9.1% vs. 17.4%, *p* < 0.05). Thus, 20-mg dose was found to be most effective, but the cumulative (40 mg, 29.2%; 60 mg, 28.1%) and 12-month incidences of PC (40 mg, 14.3%; 60 mg, 13.0%) were lower for each toremifene dose than those for the placebo. In addition, in a 3-year phase III, double-blinded, multicenter trial, 1590 men with HGPIN and negative PC findings on the prostate biopsy were randomly assigned to a toremifene citrate (20 mg) treatment or placebo group, with equal number of patients in each group [81]. Cancer was detected in 34.7% and 32.3% of the men in the placebo and treatment groups, respectively, without any difference (*p =* 0.39, log-rank test) in PC-free survival. The 3-year Kaplan–Meier PC-free survival estimate was 54.9% (99% CI, 43.3–66.5%) in the placebo group and 59.5% (99% CI, 48.1–70.9%) in the treatment group. Some previous studies had focused on adverse events associated with ADT, including osteoporosis and lipid metabolism [82,83]. However, no study has yet demonstrated the inhibitory effect for PC or for serum PSA changes.

Raloxifene, an ER agonist in the bone tissue [73], has been developed for osteoporosis treatment in women, which demonstrated some tumor-inhibitory effects against CRPC in a pilot study [84]. Raloxifene inhibited androgen-independent PC growth in 5 of 13 patients (28%). Another investigation was conducted using a combination of raloxifene and bicalutamide to evaluate treatment toxicity [85], in which 18 men with CRPC were administered a combination of bicalutamide (50 mg) and raloxifene (60 mg) over 28-day cycles. Although none of the patients required dose reduction, the resultant clinical benefit was limited. Only 4 of the 18 patients experienced >50% PSA reduction, with a median PFS of 1.9 months (1.8–2.8 months).

Fulvestrant is a pure estrogen antagonist with no agonist activity [86,89]. Fulvestrant was administered via intramuscular injection at a dose of 500 mg on day 0, followed by 250 mg on day 14, day 28, and monthly thereafter in 20 patients with CRPC [86]. Median time to progression was 4.3 months, and median OS was 19.4 months. No patient showed >50% PSA reduction or any radiological improvement.

Only one study has been conducted in patients with treatment-naïve PC [87], in which 164 patients were randomized to oral GTx-758, ERα agonist, 1000 mg/day, 2000 mg/day, or leuprolide depot. Although leuprolide reduced the total testosterone level to <50 ng/dL in most patients compared with GTx-758, GTx-758 was superior in lowering the free testosterone level and PSA. GTx-758 reduced the side effects of estrogen deficiency, such as hot flushes, bone loss, and insulin resistance, but increased the incidence of venous thromboembolic events. Therefore, the oncological outcomes of SERMs have not yet been comprehensively investigated for use in patients with treatment-naïve PC.

We thus hypothesized that additional SERMs may prolong the durability of ADT because androgen and estrogen signaling drive PC progression. In our study, we conducted a prospective randomized clinical phase IIA trial to investigate the effects of SERMs (toremifene and raloxifene) in combination with ADT in treatment-naïve bone metastatic PC [88]. Men with treatment-naïve bone metastatic PC were randomly assigned into receive ADT, toremifene (60 mg) plus ADT (TOPADT), or raloxifene (60 mg) plus ADT (RAPADT). A total of 15 men, 5 each, were allocated to one of the three treatment arms. The basal serum PSA level was 198 ng/mL (median; range, 30–8428). Bone metastases were graded as 1 (n = 11), 2 (n = 3), and 3 (n = 1) depending on the extent of the disease. During the median follow-up period of 1370 days (range, 431–1983), BCR occurred in 3, 0, and 2 men in the ADT, TOPADT, and RAPADT groups, respectively. The 5-year BCR-free rate was 30%, 100%, and 53% in the ADT, TOPADT, and RAPADT groups, respectively (*p* = 0.04, ADT vs. TOPADT; *p* = 0.48, ADT vs. RAPADT; and *p* = 0.12, TOPADT vs. RAPADT). Although ERβ agonists were expected to have tumor-inhibitory effect, the study did not prove a distinct tumor-inhibitory effect. Further additional ERβ agonists are warranted with respect to their potential role in the inhibition of human PC. Thus, we for the first time demonstrated clinical benefits of EAB in patients with treatment-naïve bone metastatic PC.

To date, anti-cancer effects of EAB have not yet been completely investigated in treatment-naïve PC patients. We assumed that the concurrent use of SERMs can prolong the duration of ADT efficacy in treatment-naïve men with bone metastatic PC. In the future, additional clinical trials with larger cohorts will be warranted to confirm our promising phase IIA findings. Furthermore, functional investigations are required to clarify the mechanism underlying EAB in treatment-naïve PC.

## 7. Molecular Diagnosis for Precision Medicine

Determination of the therapeutic strategy for breast cancer depends on the expression patterns of ERα, progesterone receptor, and human epidermal growth factor receptor 2 (HER 2) in needle biopsy samples [90]. Therapeutic strategies for non-small cell lung cancer are based on mutation of epidermal growth factor receptor (EGFR), Kirsten murine sarcoma virus (KRAS), or Anaplastic lymphoma kinase (ALK) [91]. However, pretreatment diagnosis using the estimated gene expression is not yet prevalent in patients with PC. Molecular diagnosis using immunohistochemistry; fluorescent in situ hybridization; and DNA, RNA, and/or micro-RNA analyses have been discussed in recent articles [92,93,94,95,96,97,98,99,100]. Studies in which gene sets of SC-like cells, micro-RNA, or cell-cycle progression markers reflect more aggressive diseases are limited to localized PC. A recent study has revealed that of the analysis of mutations, such as *ATM Serine/Threonine Kinase (ATM)* and *Breast Cancer 1/2* (*BRCA1/2)*, or the expression of specific genes can indicate the risk, metastatic potential, or radio sensitivity of PC [100,101]; however, investigations regarding metastatic PC through molecular diagnosis are limited.

We have previously defined an AR transcriptional network in PC cells using Chromatin immunoprecipitation on chip (ChIP–chip) and 5′-cap analysis of gene expression (CAGE) analyses [102,103] as well as functional analysis of AR-related genes, including *Amyroid precursor protein* (*APP*), Octamer-Binding Transcription Factor 1 (*Oct1*), *Tripartite Motif Containing 36 (TRIM36*), and *Forkhead Box P1* (*FOXP1*), in PC [104,105,106]. Following experiments revealed the association between BCR and the expression of *AR*, *ERα*, *Sox2*, *CRP*, and *Her2* as well as the association between CSS and the expression of *AR*, *Oct1*, *TRIM36*, *Sox2*, *Klf4*, *c-Myc*, and *ERα* in patients with metastatic PC using laser microdissection technique (LMD) [107].

**Figure 2:**
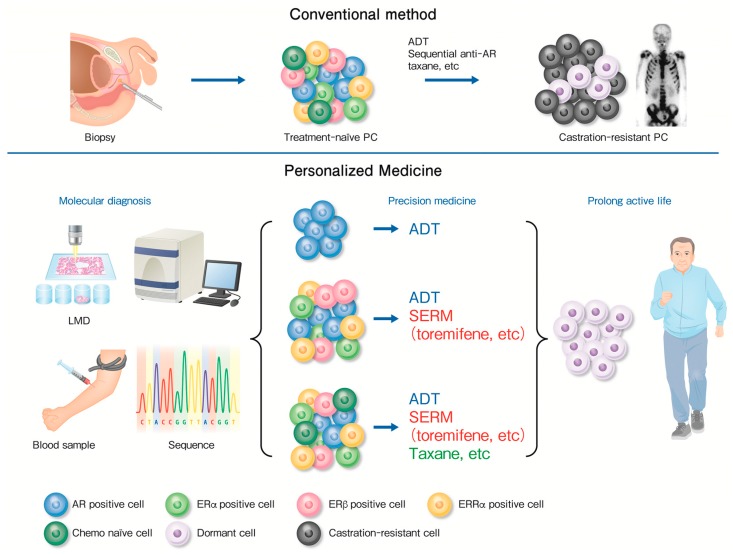
Time course of advanced prostate cancer.

Various nuclear receptors; their related co-regulators; and miR, including ARs, ERα, ERβ, and ERRs participate in the development or regulation of metastatic PC. Here, we proposed a new therapeutic strategy for metastatic PC (Figure 2). In summary, some patients with homogeneous PC cells sensitive to ADT alone, while others with heterogeneous PC cells, including ERα, ERβ, and ERRα, were eligible for EAB. In conclusion, we assume that an initial precision medicine induces dormancy in PC cells. In the future, there is a scope of precision medicine contributing to a longer and active life in patients with metastatic PC through the use of LMD of biopsy specimens, genomic analysis, or liquid biopsy of blood samples.

In classical pathways down-stream genes of AR, ER, or ERR are activated through binding AR, ER, or ERR with androgen-responsive elements (ARE), estrogen-responsive elements (ERE), or estrogen-related receptor elements (ERRE), respectively. AR is directly regulated by micro-RNA (miR)s. ERα and ERRα contribute prostate cancer (PC) progression via *phosphatidylinositol-3 kinase (Pl3K)*, *mitogen-activated protein kinase* (*MAPK*) and *Vascular Endothelial Growth Factor A* (*VEGF-A*), *Wnt Family Member 5A* (*WNT5A*), and *Transforming Growth Factor Beta 1* (*TGFβ1*) VEGF/WNT5A/TGF pathways, respectively. Estradiol and GS-1405, ERβ agonist, suppress *Forkhead Box O1* (*FOXO1*) and *Platelet Derived Growth Factor Subunit A* (*PDGFA*) expression levels through ERβ and Kruppel Like Factor (KLF) 5 pathways, and inhibit PC growth via apoptosis, anoikis, and angiogenesis. ERβ and NFκB also regulate PC activating *Interleuikin (IL)-12*, *Growth differentiation gene (GDF)-1*, *IL-8*, and *Receptor-like tyrosine kinase (RYK)*.

Conventionally most of all patients with advanced prostate cancer (PC) receive androgen-deprivation therapy (ADT) after initial diagnostic prostate biopsy irrespective of PC cell type. Consequently PC become castration resistant status with distant metastasis.

Precision or personalized medicine can separate patients according to types of PC cells; AR positive, AR, ERα, ERβ, and ERRα positive, or chemo-naïve type. Precision medicine changes advanced PC cells to dormant cells and provide patients with long and active life.

## Figures and Tables

**Table 1:** Clinical trials of selective estrogen receptors modulators for prostatic diseases.

Years (References)	Subjective	Objective	Design	Treatments	Number	Results
2001 [78]	CRPC	Cancer Control	Experimental	ADT+ Toremifene 300–640 mg/m^2^	15	No cancer inhibitory effect
2006 [79]	HGPIN	Cancer prevention	RCT	Toremifene 20, 40, 60 mg	514	Cancer prevention in 20 mg group
2008 [80]	PC	Osteoporosis prevention	RCT	Toremifene 80 mg	197	Increased bone density
2008 [82]	PC	Lipid profile improvement	RCT	Toremifene 80 mg	1389	Decreased T Cho, LDL, HDL, TG
2013 [81]	HGPIN	Cancer prevention	RCT	Toremifene 20 mg	1467	Not significant cancer prevention
2006 [84]	CRPC	Cancer Control	Experimental	Raloxifene 60 mg	13	Partial effect (5 of 13 patients)
2017 [85]	CRPC	Cancer Control	Experimental	Raloxifene 60 mg + Bicaltamide 50 mg	18	Partial effect (4 of 18 patients)
2008 [86]	CRPC	Cancer Control	Experimental	Fulvestrant 500 mg, 250 mg	20	No patients reduced >50% PSA reduction
2015 [87]	Hormone naïve PC	Testosterone reduction	RCT	ADT, ADT+ GTx-758 1000 mg, or ADT+ GTx-758 2000 mg	164	Superior testosterone reduction in GTx-756 group
2015 [88]	Hormone naïve PC	Cancer Control	RCT	ADT, ADT+ Toremifene 60 mg, or ADT+ Raloxifene 60 mg	15	ADT+ toremifene significantly improved BCR

PC; Prostate cancer, CRPC; Castration-resistant prostate cancer, ADT; Androgen deprivation therapy, HGPIN; high-grade prostatic intra-epithelial neoplasia, RCT; randomized clinical trial, T Cho; Total cholesterol, LDL; Low density lipoprotein, HDL; High density lipoprotein, TG; Total glyceride, BCR; Biochemical recurrence.
